# Modulation of the Antioxidant Defense System by Exogenous l-Glutamic Acid Application Enhances Salt Tolerance in Lentil (*Lens culinaris* Medik.)

**DOI:** 10.3390/biom11040587

**Published:** 2021-04-16

**Authors:** Jannatul Fardus, Md. Shahadat Hossain, Masayuki Fujita

**Affiliations:** Laboratory of Plant Stress Responses, Faculty of Agriculture, Kagawa University, Ikenobe 2393, Miki-Cho, Kita Gun, Kagawa 761-0795, Japan; jannatulsau11@gmail.com (J.F.); shahadatsau24@gmail.com (M.S.H.)

**Keywords:** salt stress, ROS, oxidative damage, ion homeostasis, antioxidant defense system, amino acid

## Abstract

Salt stress greatly disturbs the growth, morpho-physiological, and biochemical performance of plants. However, different physiological processes and acclimation mechanisms can be induced under stress, while some of them can be modulated by the appropriate chemical stimulus. The objective of this study was to evaluate the impact of exogenous pretreatment with 10 mM l-glutamic acid (l-Glu) on the physiological and biochemical parameters of lentil (*Lens* *culinaris* Medik.) under 110 mM NaCl stress. Salt stress inhibited the growth and reduced the photosynthetic pigment (chlorophylls and carotenoids) level, water content, and survival of lentil seedlings during recovery from the stress. Salt stress also induced oxidative damage, as indicated by higher hydrogen peroxide and malonaldehyde contents and electrolyte leakage, by interrupting the antioxidant defense system and promoting the accumulation of toxic levels of Na^+^. However, l-Glu pretreatment mitigated the salt-induced damage in lentil seedlings by reducing the accumulation of Na^+^, maintaining ion homeostasis, and increasing the activities of antioxidant enzymes (catalase and ascorbate peroxidase). As a result, salt-induced oxidative damage was reduced, seedling growth and photosynthetic pigment contents were enhanced, and the survival rate of the lentil seedlings was improved in response to salt stress, indicating an ameliorative role for l-Glu in lentil seedling growth under salt stress.

## 1. Introduction

Among the abiotic stresses experienced by crop plants, salinity is a major abiotic stress that affects about 20% of the world’s total cultivable land, estimated at around 800 million hectares. Current estimates indicate that approximately half of the present-day cultivable land will be unsuitable for cultivation by the middle of the 21st century due to salinity, posing a serious threat to global crop production [1].

Exposure of plants to a soluble salt in excess of the threshold level (4 dS m^−1^ or higher) causes osmotic stress and reduces plant growth [2]. Ionic stress follows, with the increasing accumulation of toxic ions, mainly Na^+^ and Cl^−^, and this leads to necrosis and death of the leaves from older to younger [1]. Both osmotic stress and ionic toxicity inhibit photosynthesis, disrupt ion homeostasis, inactivate vital enzymes, and increase the accumulation of reactive oxygen species (ROS), such as singlet oxygen (^1^O_2_), superoxide radical (O_2_·^−^), hydrogen peroxide (H_2_O_2_), and hydroxyl radical (OH·) [3,4]. ROS reduce photosynthetic ability, denature proteins, and promote the peroxidation of lipids, with subsequent death of the plants [1].

Under optimal growth conditions, a minimal level of ROS is generated in different plant organelles [5,6]. However, the generation of ROS increases in response to stress and causes oxidative bursts in the organelles of plants [7]. Plants cope with the damage caused by salt stress by activating antioxidant defense mechanisms that involve both non-enzymatic components (ascorbic acid (AsA), reduced glutathione (GSH), and oxidized glutathione (GSSG)) and enzymatic components (catalase (CAT), ascorbate peroxidase (APX), monodehydroascorbate reductase (MDHAR), dehydroascorbate reductase (DHAR), glutathione reductase (GR), glutathione peroxidase (GPX), and glutathione *S*-transferase (GST)) working in concert [7,8,9,10].

Evidence suggests that the accumulation of osmolytes (such as proline (Pro), glycine-betaine, and sugar alcohols), inhibition of Na^+^ uptake, transport of Na^+^ from the root to the shoot, restriction of K^+^ leakage, and detoxification of ROS by the antioxidant defense system work synergistically to mitigate salt-induced damage in plants [1,11,12]. A few technologies have been established to enhance tolerance in plants to abiotic stresses, including salt stress [13,14,15,16]. For example, the use of chemicals can alleviate stress-induced damage without resorting to genetic alterations of crop plants [17,18]. Numerous studies now suggest an enhancement of salt stress tolerance in plants by several different chemicals [19,20,21,22], including gibberellic acid, salicylic acid, and γ-aminobutyric acid, in wheat, maize, and rice [23,24,25,26]. In recent times, a non-essential amino acid, glutamate (Glu), was found to play a vital role in plant growth and development processes (seed germination and pollen and root architecture) and in stress acclimation [27,28]. La et al. [27] suggested that exogenous application of Glu could enhance drought tolerance in radish. These findings suggested to us that l-Glu might increase salt tolerance in plants. However, the role of l-Glu in enhancing salt stress tolerance in important crop plants has not yet been investigated.

One particularly significant crop among the other pulse crops grown in Bangladesh is lentil (*Lens culinaris*) [29,30]. Lentil remains the most essential legume crops in many countries of the world due to its high protein content [31]. In addition, lentil plants can fix nitrogen from the atmosphere in the soil through root nodules [32]. However, lentil plants are sensitive to salt stress; therefore, increasing soil salinity has reduced the production of lentils worldwide and threatens the protein intake of large segments of the human population through its effects on soil health [33].

In the present study, we investigated the effect of exogenous application of l-Glu in mitigating salt-induced damage in lentil, as a less well-researched but economically important crop. We observed the plant growth, oxidative damage, and activity of different antioxidants during recovery from salinity stress. To the best of our knowledge, this is the first report showing the potential for the use of l-Glu to enhance salt stress tolerance in lentil.

## 2. Materials and Methods

### 2.1. Plant Growing Conditions and Treatment

Uniform seeds of lentil (*Lens culinaris* Medik cv. BARI Masur-7) were surface sterilized for 5 min using 70% ethanol, then dipped in distilled water for 24 h and placed on moistened six layers of paper towels in the Petri dishes and kept in dark conditions for 72 h. Keeping 40 germinated seedlings per Petri plate, Petri plates were then transferred into the growth chamber under a photon flux density of 350 μmol m^−2^ s^−1^ with continuous illumination and 25 ± 1 °C temperature. After 24 h, two sets of Petri plate were provided with 10 mM l-Glu along with Hyponex nutrient solution [34]. Another two sets of Petri plate were provided with only Hyponex (Tokyo, Japan) nutrient solution. Then 6-day-old seedlings were treated with NaCl (110 mM) with or without l-Glu pretreatment. The dose of l-Glu and salt stress was selected based on our preliminary trial and former report (Figures S1a–d and S2a–d) [35]. After 2 days of stress treatment, seedlings were allowed to recover by supplying nutrient solution only. Thus, the treatments were control (without NaCl), 10 mM l-Glu, 110 mM NaCl (S), and 110 mM NaCl with 10 mM l-Glu (S + l-Glu), and each treatment had three replications. Finally, physiological, and biochemical parameters were measured from the seedlings.

### 2.2. Assessment of Survival Percentage

We counted the fully recovered seedlings after 7 days of recovery for evaluating survival percentage following the formula of Gong et al. [36]. The formula was survival (%) = survived seedlings × 100/total number of seedlings.

### 2.3. Determination of Shoot Fresh Weight, Root Fresh Weight, Root Fresh Weight, Root Dry Weight and Water Content

To estimate shoot and root fresh and dry weight, 10 shoots and roots of randomly selected seedlings were separated, and then fresh weight of shoot (SFW) and root (RFW) were determined after extra moisture was removed using a paper towel. Later, the same 10 seedlings were kept in a dryer for 48 h with 80 °C temperature until showing constant weight and then the dry weight of three replications of shoot (SDW) and root (RDW) was recorded. Thereafter, water content (WC) was estimated by following the formula: WC (%) = ((FW − DW)/FW) × 100.

### 2.4. Evaluation of Chlorophyll and Carotenoid Content

A leaf sample (0.1 g) was collected and put in a test tube containing 10 mL of DMSO. For the efficient extraction of chlorophylls (Chl) and carotenoids (Car), the test tube was placed in a water bath for heating for about 1 h with 65 °C temperature. After 1 h, the sample was kept in a room for cooling and then absorbance was measured at 665 and 649 nm wavelengths followed by the formula of Wellburn [37]. Following Wellburn [37], the absorbance of carotenoid (Car) content was measured at 480 nm wavelength. The expressing unit of Chls and Car was mg g^−1^ FW.

### 2.5. Measurement of Proline Content and Electrolyte Leakage

According to Bates et al. [38], proline (Pro) content was measured from the shoot of each sample and expressed as µmol g^−1^ FW. The assessment of electrolyte leakage (EL) was done by following the method of Dionisio-Sese and Tobita [39].

### 2.6. Estimation of Malonaldehyde, Other Aldehyde and Hydrogen Peroxide

A total of 3 mL of ice-cold 5% TCA (trichloroacetic acid) was used for homogenizing a 0.5 g shoot of plant sample while using a mortar and pestle and then centrifuged for 15 min at 11,500× *g*. After centrifugation, 1 mL of supernatant was mixed with 4 mL of TBA (thiobarbituric acid) and heated for 30 min. After heating, the sample was kept in an ice box for cooling and then centrifuged again for 10 min. Finally, this supernatant was used for estimating malonaldehyde (MDA) as the difference between absorbance at 532 and at 600 nm, while the other aldehyde contents also determined the absorbance at 455 nm [40,41]. MDA and other aldehyde contents were calculated using co-efficients of 155 mM^−1^ cm^−1^ and 0.457 × 10^5^ M^−1^ cm^−1^, respectively. Measurement of hydrogen peroxide (H_2_O_2_) was done according to Yang et al. [42] using the absorbance at 390 nm.

### 2.7. Assessment of Reduced Ascorbate, Reduced Glutathione, and Oxidized Glutathione Content

The plant sample (0.5 g) was centrifuged for 15 min at 11,500× *g* after homogenizing with 3 mL of 5% TCA. The supernatant was collected from the centrifuged sample, which was then used for the determination of ascorbate (AsA), total glutathione and oxidized glutathione (GSSG) followed by the method of Noctor et al. [43] through neutralizing with 0.5 M K-P (potassium phosphate) buffer at pH 7.0 and expressed as µmol g^−1^ FW and nmol g^−1^ FW.

### 2.8. Measurement of Total Soluble Protein

The concentration of total soluble protein was assayed and computed through using bovine serum albumin (BSA) as a standard of protein followed by the method of Bradford [44].

### 2.9. Estimation of the Activity of Enzyme

Firstly, 0.5 g of lentil shoots from each sample was taken in a mortar and pestle and homogenized with 50 mM buffer containing ascorbate (1 mM), KCl (100 mM), β-marceptoethanol (5 mM) and glycerol (10% *w*/*v*). After homogenizing, the sample was centrifuged at 11,500× *g* for 15 min at 4 °C and the supernatant then transferred in an Eppendorf tube which was later used for estimating soluble protein concentration and the activity of enzymes.

The activity of catalase (CAT, EC:1.11.1.6) was measured as the absorbance at 240 nm with the addition of H_2_O_2_ and enzyme described by Noctor et al. [43] and computed using 40 mM^−1^ cm^−1^ extinction coefficient; the expressing unit was µmol min^−1^ g^−1^ protein.

Ascorbate peroxidase (APX, EC:1.11.1.11) activity was estimated using extracted enzyme which mixed with K-P buffer (50 mM) having pH 7.0, AsA (0.5 mM), H_2_O_2_ (0.1 mM) and EDTA (0.1 mM) at an absorbance of 290 nm and expressed as nmol min^−1^ mg^−1^ protein [43].

To assess the activity of monodehydroascorbate reductase (MDHAR, EC:1.6.5.4), Tris-HCl buffer (50 mM) at pH 7.5, AsA (2.5 mM) and NADPH (0.2 mM) were added with extracted enzyme and measured the absorbance at 290 nm. The unit of MDHAR was nmol min^−1^ mg^−1^ protein and calculated using 6200 M^−1^ cm^−1^ as an extinction co-efficient [43].

According to the formula of Noctor et al. [43], dehydroascorbate reductase (DHAR, EC:1.8.5.1) was measured as the absorbance at 265 nm after the addition of K-P buffer (50 mM) at pH 7.0, GSH (2.5 mM) and DHA (0.1 mM) with extracted enzyme and expressed as nmol min^−1^ mg^−1^ protein.

The activity of glutathione reductase (GR, EC:1.6.4.2) was assayed by following the method of Noctor et al. [43], where extracted enzyme was mixed with K-P buffer (20 mM) at pH 7.0, EDTA (1 mM), GSSG (0.1 mM) and NADPH (1.35 mM), and absorbance was measured at 340 nm and computed by using a 6200 M^−1^ cm^−1^ extinction coefficient. The activity was expressed as nmol min^−1^ mg^−1^ protein.

To determine the activity of glutathione *S*-transferase (GST, EC:2.5.1.18), extracted enzyme was mixed with Tris-HCl buffer (100 mM) at pH 6.5, GSH (1.5 mM) and 1-chloro-2,4-dinitrobenzene (CDNB, 1 mM) and the absorbance at 340 nm. The activity was calculated by using 9600 M^−1^ cm^−1^ as the extinction coefficient. The activity was expressed as nmol min^−1^ mg^−1^ protein [45].

Glutathione peroxidase (GPX, EC:1.11.1.9) activity was estimated through using K-P buffer (100 mM) at pH 7.0, EDTA (1 mM), NaN_3_(1 mM), NADPH (0.12 mM), GSH (2 mM), GR (1 unit) and H_2_O_2_ (0.6 mM) with extracted enzyme by measuring the absorbance at 340 nm and expressed as nmol min^−1^ mg^−1^ protein.

### 2.10. Determination of Methylglyoxal Level

Shoots (0.25 g) of lentil seedlings were homogenized with 2.5 mL 5% perchloric acid (PCA) using a mortar and pestle and then centrifuged at 11,000× *g* for 10 min. The supernatant was mixed with charcoal and centrifuged again. The clarified supernatant was neutralized using saturated Na_2_CO_3_ and used for the estimation of methylglyoxal (MG) through the addition of NaH_2_PO_4_ (sodium hydrogen phosphate) and N-acetyl-L-cysteine. The absorbance was measured at 288 nm for MG determination.

### 2.11. Statistical Analysis

XLSTAT v.2020 software (Addinsoft, Paris, France) using Fisher’s least significant difference (LSD) at 5% probability (*p* ≤ 0.05) used to evaluate the comparable mean difference of three replications and the analysis of variance (ANOVA).

## 3. Results

### 3.1. l-Glu Improved the Phenotypic Appearance and Survival of Lentil Seedlings Grown under Salinity Stress

**Figure 1:**
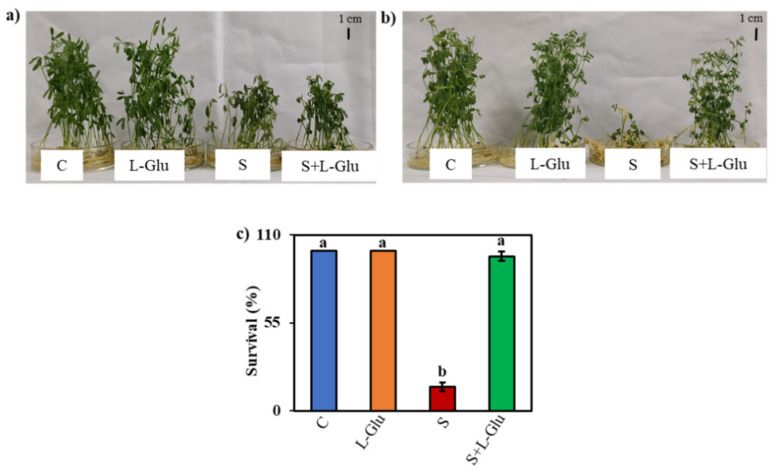
Effect of l-Glutamic acid (l-Glu) on (**a**) the phenotypic appearance of lentil seedlings after 2 days of recovery, (**b**) the phenotypic appearance of lentil seedlings after 7 days of recovery and (**c**) the survival percentage of lentil seedlings after 7 days of recovery. The treatments were control (C), 10 mM l-Glu, 110 mM NaCl (S) and 110 mM NaCl + 10 mM l-Glutamic acid (S+ l-Glu). The above mean (± SE) was calculated from three replications. Values of different letters indicate statistically significant differences at *p* ≤ 0.05 (Fisher’s LSD test).

The effect of salt stress and the role of l-Glu in mitigating salt-induced damage in lentil seedlings were investigated by initially exposing the seedlings to 110 mM salt stress with or without different doses of l-Glu ranging from 0.3 to 20 mM (Figure S1a–d). We ultimately selected 10 mM l-Glu for further work, as this dose gave a better recovery of the plant phenotype following 110 mM NaCl stress (Figure S1a–d). The dose of salt stress was selected from a range of 75–150 mM NaCl (Figure S2a–d). The highest dose of NaCl (150 mM) showed lower survival than the lower doses in response to l-Glu pretreatment (Figure S2d). We also checked the effects of other amino acids in the same concentration of l-Glu (10 mM), such as l-glutamine, l-glycine, l-asparagine, l-methionine, and l-cysteine, under salt-stress conditions, but l-Glu pretreatment gave a better phenotypic appearance and survival performance compared to the other amino acids (Figure S3a,b). The stressed plants became stunted and wilted and some leaves turned yellowish in color, but the plants pretreated with l-Glu were healthier and greener as they recovered from the imposed salt stress (Figure 1a,b). As the recovery period progressed, more seedlings failed to recover from the salt stress in the absence of the l-Glu treatment. Approximately 97% of the seedlings survived the saline treatment when pretreated with l-Glu vs. approximately 15% of plants that survived when exposed to 110 mM salt without l-Glu pretreatment (Figure 1c).

### 3.2. l-Glutamic Acid Improved the Growth and Water Content and Reduced the Proline Content Of Lentil Plants Exposed to Salt Stress

Salt stress has many detrimental effects on plants; therefore, we evaluated the protective role of l-Glu pretreatment by measuring growth and water status parameters, such as SFW, SDW, WC, and Pro content (Table 1). The SFW and SDW of salt-stressed plants decreased by 50% and 23%, respectively, in comparison with the control. Conversely, l-Glu pretreatment elevated the SFW and SDW by 38% and 7%, respectively, compared with salt-stressed seedlings without l-Glu pretreatment (Table 1). The WC declined by 11% under salt-stress conditions, but it was increased by 8% after the application of l-Glu (Table 1). The Pro content increased by 374% compared to unstressed control plants in response to the 110 mM salt treatment, but pretreatment with l-Glu reduced the amount of proline (by 42%) in plants under salinity stress compared to salt-stressed plants (Table 1).

### 3.3. l-Glu Protected Photosynthetic Pigments under Saline Stress

**Figure 2:**
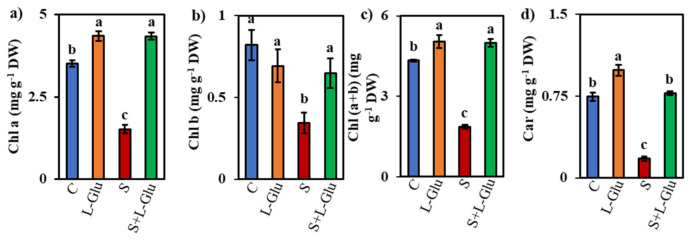
Effect of l-Glutamic acid (l-Glu) on photosynthetic pigment (**a**) Chlorophyll a (Chl a), (**b**) Chlorophyll b (Chl b), (**c**) Chlorophyll (a + b) (Chl (a + b)) and (**d**) the Carotenoid (Car) content of lentil seedlings under salt stress and subsequent recovery. The above mean (± SE) was calculated from three replications. Values of different letters indicate statistically significant differences at *p* ≤ 0.05 (Fisher’s LSD test).

Measurements of photosynthetic pigments, such as Chl a, Chl b, Chl (a + b), and Car, in leaves of lentil plants under salt stress revealed declines from the unstressed values Chl a (1.52 mg) Chl b (0.34 mg), Chl (a + b) (1.9 mg) and Car (0.17 mg) of 57%, 58%, 57%, and 76%, respectively (Figure 2a–d). However, l-Glu application alleviated salt stress since pigment composition in S + l-Glu was 186%, 89%, 168% and 336% of that of the control for Chl a, Chl b, Chl (a + b) and Car, respectively (Figure 2a–d).

### 3.4. l-Glu Decreased Oxidative Damage in Lentil Plants

**Figure 3:**
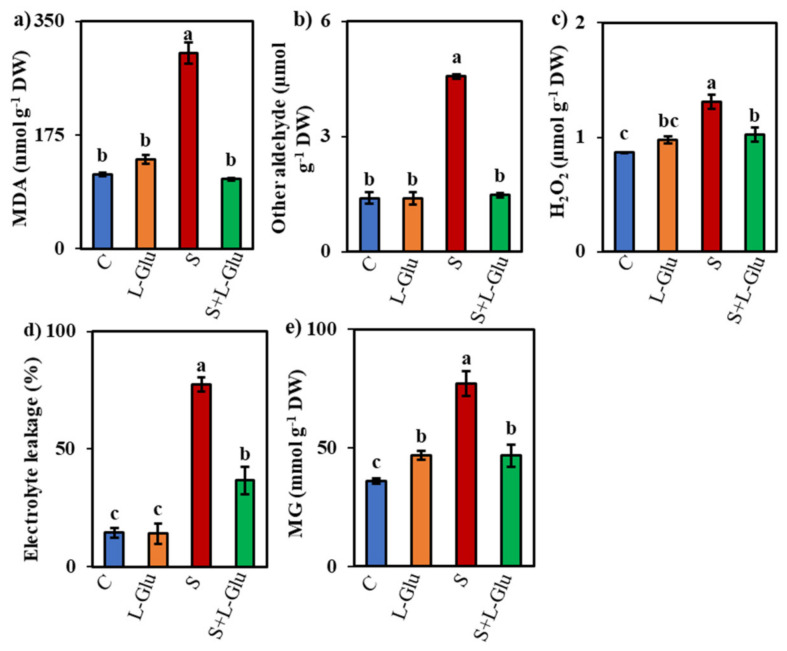
Effect of l-Glutamic acid (l-Glu) on the (**a**) malonaldehyde (MDA), (**b**) other aldehyde, (**c**) hydrogen peroxide (H_2_O_2_), (**d**) electrolyte leakage and (**e**) methylglyoxal (MG) content of lentil seedlings under salt stress and recovery conditions. The above mean (± SD) was calculated from three replications. Values of different letters indicate statistically significant differences at *p* ≤ 0.05 (Fisher’s LSD test).

Lipid peroxidation is a major sign of oxidative damage in plants and can be measured by assessing the MDA content. Salt stress increased the MDA content by 164% compared with unstressed control plants, but l-Glu-pretreatment suppressed this salt-stress-induced increase in MDA content by 65% (Figure 3a). Compared with the unstressed controls, the content of other aldehydes, H_2_O_2_ content, and EL and MG content increased by 229%, 51%, 434%, and 114%, respectively, under salt stress (Figure 3b–e). Conversely, in l-Glu-pretreated seedlings, these indicators declined by 68%, 22%, 53%, and 40%, respectively, compared with salt-stressed seedlings (Figure 3b–e).

### 3.5. l-Glu Impact on Non-Enzymatic and Enzymatic Antioxidants under Salinity Stress

**Figure 4:**
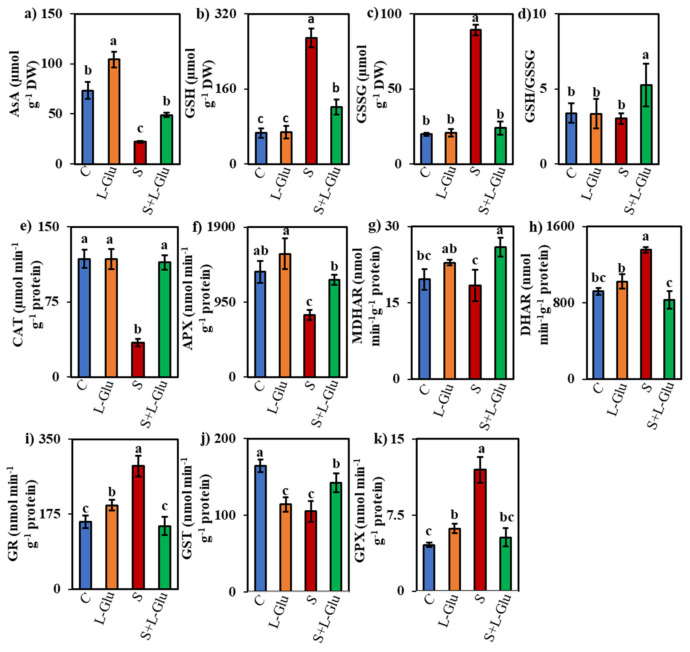
Effect of l-Glutamic acid (l-Glu) on the content of non-enzymatic antioxidants (**a**) ascorbate (AsA), (**b**) reduced glutathione (GSH), (**c**) oxidized glutathione (GSSG) and (**d**) GSH/GSSG; activity of enzymatic antioxidants (**e**) catalase (CAT), (**f**) ascorbate peroxidase (APX), (**g**) monodehydroascorbate reductase (MDHAR), (**h**) dehydroascorbate reductase (DHAR), (**i**) glutathione reductase (GR), (**j**) glutathione *S*-transferase (GST) and (**k**) glutathione peroxidase (GPX) of lentil seedlings under salt stress and recovery conditions. The above mean (± SE) was calculated from three replications. Values of different letters indicate statistically significant differences at *p* ≤ 0.05 (Fisher’s LSD test).

We investigated the effect of l-Glu on non-enzymatic and enzymatic antioxidants in response to salt stress by measuring the non-enzymatic antioxidant content ((AsA, GSH, and GSSG) and GSH/GSSG ratio) and enzyme activities (CAT, APX, MDHAR, DHAR, GR, GST, and GPX). The AsA content increased in the l-Glu-pretreated seedlings under both control and salt-stress conditions by 42% and 123%, respectively, when compared to untreated control and salt-stressed seedlings, respectively (Figure 4a). However, AsA contents were 70% lower in plants exposed to 110 mM salt than in the unstressed controls (Figure 4a). Conversely, the GSH and GSSG contents were enhanced by 305% and 353%, respectively, whereas the GSH/GSSG ratio was reduced by 11% under the salt-stress condition compared with the unstressed control (Figure 4b–d). l-Glu pretreatment reduced the GSH and GSSG content by 55% and 73%, respectively, compared with untreated salt-stressed seedlings, whereas l-Glu pretreatment increased the GSH/GSSG ratio by 74% under salinity stress compared with the untreated salt-stressed seedlings (Figure 4b–d).

Salt stress decreased the activity of CAT and APX by 71% and 41%, respectively, compared to the unstressed controls (Figure 4e,f). However, l-Glu pretreatment enhanced the CAT and APX enzyme activity by 232% and 57% compared with the untreated salt-stressed seedlings (Figure 4e,f). MDHAR activity was increased by 17% and 41% in l-Glu-treated seedlings under control and salt-stress conditions, respectively, when compared with the control and salt-stress conditions in the absence of l-Glu pretreatment (Figure 4g). The DHAR activity increased (by 47%) under salinity stress, but l-Glu pretreatment suppressed this increase by 39% under the salt-stress condition (Figure 4h).

The activities of GR and GPX increased by 83% and 162%, respectively, in salt-stressed seedlings compared with the unstressed control (Figure 4i,j). However, l-Glu pretreatment suppressed this increase by 49% and 56%, respectively, compared with the untreated salt-stressed plants (Figure 4i,j). By contrast, the GST activity increased by 36% in response to l-Glu pretreatment compared to the untreated salt-stressed plants (Figure 4k).

### 3.6. l-Glu Regulated Ion Homeostasis during Salt Stress

We further examined the role of l-Glu on ion homeostasis in response to salt stress by determining the Na^+^, K^+^, Ca^2+^, and Mg^2+^ contents in the leaves, shoots, and roots of lentil seedlings. l-Glu pretreatment resulted in lower plant leaf, shoot, and root levels of Na^+^ (33%, 32%, and 33% lower, respectively) and higher K^+^ levels (3%, 71%, and 67%, respectively) compared to untreated salt-stressed plants (Table 2). However, the salt-stressed seedlings showed markedly higher leaf, shoot, and root Na^+^ contents and lower K^+^ contents compared with the unstressed control plants (Table 2). The leaf, shoot and root Na^+^/K^+^ ratios were also markedly higher in the salt-stressed seedlings than in the controls. Pretreatment with l-Glu decreased the ratios of Na^+^/K^+^ by 35%, 61%, and 60% for the leaves, shoots, and roots, respectively, when compared with the untreated salt-stressed seedlings (Table 2). Salt stress also decreased the leaf, shoot, and root contents of Ca^2+^ (by 71%, 80%, and 56%, respectively) and the shoot and root contents of Mg^2+^ (by 58% and 98%, respectively) compared with the unstressed controls (Figure S4a–f). l-Glu pretreatment suppressed the decline in leaf, shoot, and root Ca^2+^ and Mg^2+^ levels in response to salt stress (Figure S4a–f).

### 3.7. Correlation Analysis

We used correlation analysis to identify important factors for salt tolerance in lentil plants. The accumulation of Na^+^ was positively correlated with the oxidative damage markers (MDA, H_2_O_2_, EL, MG, and other aldehydes) but was negatively correlated with growth parameters, survivability, and photosynthetic pigment levels (Figure S5). Among the components of the antioxidant defense pathway, CAT, APX, and AsA showed a negative correlation with the oxidative damage markers (Figure S5).

## 4. Discussion

Soil salinity severely disturbs the plant morphological, physiological, biochemical, and metabolic processes, including enzymatic activity, photosynthesis, and cell membrane integrity [46]. These disturbances in vital processes in plants under salt stress result in crop yield decreases and pose a serious threat to global food security. Thus, mitigating salt-induced damage in plants is an important research objective. Scientists have already started to use different strategies, including the use of exogenous chemicals, to mitigate salt-induced damage in different plants [47,48], but the search continues for low-cost chemicals that are both effective and ecofriendly. The results presented here indicate that l-Glu could play an important role as a low-cost chemical capable of alleviating the adverse effects of salt stress in lentil seedlings. l-Glu, as a protein amino acid, plays an important role in the growth and development of plants and in adaptation to stress [27,28]. However, the benefits of the exogenous application of l-Glu in enhancing salt stress tolerance have not been adequately explored.

In our investigation, we first determined whether l-Glu application could enhance the survival of lentil seedlings after 7 days of recovery from salt stress (Figure 1b,c). Most of the seedlings withered and failed to survive during recovery from salt stress. However, the l-Glu pretreatment enhanced the survival rate by 97% (Figure 1c), indicating that l-Glu is able to aid in overcoming the detrimental effects of salt stress. Compared to other amino acids, l-Glu was more effective at inducing salt tolerance (Figure 3a,b and Figure S2c). One clear response to salt stress was an arrest of plant growth, as indicated by declines in biomass production (shoot fresh weight and dry weight) (Table 1); these effects have been attributed to osmotic shock [1]. Our findings agreed with a previous report by Hossain et al. [49,50], who also noted a salt stress-induced growth reduction and lower survival rate in lentil seedlings. However, the pretreatment with l-Glu was able to mitigate the salt-induced losses in the shoot fresh weight and dry weight of lentil seedlings, suggesting that l-Glu reduces the effect of osmotic stress (Table 1). Sadak et al. [51] reported that a mixture of amino acids, which included l-Glu, enhanced biomass under salt-stress conditions, and they suggested a potential role for amino acids as biostimulants.

When under salt stress, plants may sense perturbations in water status and respond by synthesizing different osmolytes, most notably amino acids such as Pro or carbohydrates and their derivatives, which act as osmoprotectants [52,53]. In the present study, stressed plants accumulated more Pro in proportion to their water content (Table 1). l-Glu pretreatment reduced the accumulation of Pro in response to salt stress (Table 1), indicating that the l-Glu-pretreated seedlings experienced a lesser worsening of water status, as revealed by their higher water content compared to the salt-stressed plants (Table 1).

The photosynthetic pigment content is another important parameter that is directly correlated with plant growth and biomass [54]. Salt stress markedly reduced the levels of chlorophylls (Chl a, Chl b, Chl (a + b)) and Car (Figure 2a–d), in agreement with the findings of Qados [55], Rahman et al. [56], and Pandey and Sengar [57], who reported photosynthetic pigment damage in bean, acacia, and lentil seedlings under salt stress. The reduction in Chl (a + b) and Car content indicates that lentil seedlings failed to improve photosynthetic efficiency in response to salt stress, with further effects on plant growth and lower biomass. However, l-Glu enhanced the contents of Chl a, Chl b, Chl (a + b) and Car in both salt-stressed and unstressed seedlings (Figure 2a–d), indicating a direct involvement of l-Glu in light energy capturing and cooperation among the different pigments to increase photosynthetic performance. This result was consistent with the findings of La et al. [27], who demonstrated a Glu-mediated augmentation of total Chl and Car contents in radish seedlings under drought stress conditions.

Like other abiotic stresses, salt stress elevates ROS production and causes instability in the antioxidant defense system that, in turn, results in severe oxidative damage in plants [58]. In our study, we measured the content of MDA, other aldehydes, and H_2_O_2_, as well as EL and MG, to understand the level of oxidative damage in salt-stressed lentil plants (Figure 3a–e). We found increases in all these parameters in response to salt stress (Figure 3a–e), indicating severe oxidative damage. Our results agree with previous findings reporting increased ROS load in plants growing in saline environments [59,60,61]. Pretreatment with l-Glu rescued the lentil seedlings from ROS-induced oxidative stress by lowering the MDA and H_2_O_2_ content (Figure 3a,c) while also reducing the extent of EL (Figure 3d). These results are in agreement with previous work showing that l-Glu application decreases ROS accumulation under drought stress [27]. As a dicarbonyl compound, MG causes the denaturation of DNA, RNA, protein, and lipids by increasing ROS accumulation and inhibiting the activity of antioxidants [62,63]. MG showed an overaccumulation in salt-stressed lentils (Figure 3e), in agreement with previous reports of high MG accumulations in wheat, mustard, and mung bean seedlings under salt stress [64,65,66]. However, l-Glu pretreatment suppressed this MG content accumulation in response to salt stress (Figure 3e), suggesting that l-Glu pretreatment reduced ROS accumulation and oxidative damage by modulating the components of the antioxidant defense system.

Stress tolerance can be achieved by efficient ROS detoxification, especially through the action of enzymatic and non-enzymatic antioxidants, which increased plant survival under stress conditions by protecting plants from oxidative damage [67,68]. AsA and GSH are two vital non-enzymatic components of the plant antioxidant defense system, where they work as promoters of redox buffering and as suppliers of electrons to APX and GPX enzymes [69,70,71]. AsA is regenerated by the enzyme pair MDHAR and DHAR. Conversely, GR helps to maintain the GSH pool by regenerating GSH from GSSG by the activity of GST following glutathione oxidation and by managing the redox balance of plant by maintaining a stable GSH/GSSG ratio [72]. In the present study, we observed lower CAT and APX activities and AsA contents in stressed lentil plants (Figure 4a,e,f). This may explain the higher H_2_O_2_ accumulation observed under salt stress, as CAT and APX activities convert H_2_O_2_ to water. Tepe and Aydemir [73] also reported a downregulation of CAT and APX in lentils under salt stress, in line with our findings. L-Glu application prevented the suppression of CAT and APX activities under salt stress (Figure 4e,f), suggesting that l-Glu application can modulate both the enzymatic and non-enzymatic components of the antioxidant defense pathway under stress conditions. La et al. [27] reported an increase in CAT activity under drought stress following l-Glu application.

In our study, we observed increased activities of DHAR, GR, and GPX, but no change in MDHAR and GST activities under salt stress (Figure 4g–k), indicating that seedlings acclimated to the stress conditions by reducing H_2_O_2_ through GPX and by increasing AsA and GSH recycling through DHAR and GR. Different responses of the components of the antioxidant defense pathway have also been reported in rice [25], maize [74], and soybean [75]. In our study, l-Glu application decreased DHAR, GPX, and GR activities and increased the MDHAR and GST activities in the salt-stressed seedlings (Figure 4g–k). We observed different responses of the components of the antioxidant defense pathway in lentil seedlings under salt-stress conditions, suggesting a disturbance in the maintenance of ROS homeostasis. The response of the antioxidant defense pathway varies with the dose, duration, and type of stress, as well as the age and genotype of the plant species and other experimental conditions [3]. Upregulation of one or two components, instead of the full complement of the pathway, may enhance stress tolerance in plants [3]. Current evidence suggests that increasing CAT activity and AsA content are essential for salt and copper stress tolerance in lentil seedlings [34,49,50]. We observed a strong negative correlation between oxidative damage markers and CAT, APX and AsA activities (Figure S5), suggesting that increased activities of CAT, APX and AsA mediated by l-Glu play a critical role in reducing the oxidative damage under salt stress, thereby contributing to the better survival of seedlings under such conditions.

Salt tolerance in plants largely depends on the accumulation and translocation of toxic Na^+^ in different parts of plants [9,76]. Salt stress also disrupts nutrient homeostasis by interrupting the uptake and transport of K^+^, Ca^2+^, and Mg^2+^ [49,77]. Maintaining nutrient homeostasis and the Na^+^/K^+^ ratio is crucial for plant growth and survivability in saline environments [78,79]. In this study, salt-stressed seedlings had higher Na^+^ levels and Na^+^/K^+^ ratios, and lower K^+^, Ca^2+^, and Mg^2+^ levels, indicating that salt stress disturbed ion homeostasis, thereby resulting in lower biomass production (Table 2 and Figure S4a–f). Correlation analysis suggested that growth parameters and photosynthetic pigments were strongly negatively correlated with Na^+^ accumulation (Figure S5). Singh et al. [80] and Hossain et al. [50] also reported higher accumulations of Na^+^ and higher Na^+^/K^+^ ratios, as well as lower K^+^, Ca^2+^, and Mg^2+^ levels in lentil seedlings under salt stress. However, l-Glu pretreatment reduced the Na^+^ accumulation and restored the Na^+^/K^+^ ratio while also increasing the K^+^, Ca^2+^ and Mg^2+^ contents in lentil seedlings (Table 2 and Figure S4a–f). Exogenous application of amino acids reduced the Na^+^ accumulation in leaves of faba bean [51], in agreement with our results. These responses could explain the reduction in photosynthetic pigment damage and the better growth of the l-Glu-treated seedlings under salt stress. Exogenous chemicals, such as acetate, calcium chloride, glutathione, polyamines, and amino acids, are now well established as having the potential to maintain nutrient homeostasis in plants under salt stress, thereby enhancing salt tolerance [50,51,77,81,82].

## 5. Conclusions

Salt stress inhibited the growth and lowered the survival of lentil seedlings during recovery from salt stress. The application of l-Glu negated the salt-induced damage by inducing various biochemical and physiological responses, including (a) the reduction of Na^+^ accumulation and maintenance of ion homeostasis, (b) the protection of photosynthetic pigments, and (c) the reduction of oxidative stress through increased action of antioxidant defense systems (CAT, AsA, and APX). These findings imply that the pretreatment of salt-stressed plants with l-Glu could be a cost-effective method for reducing the detrimental effects of salt stress on crop growth. However, this study evaluated the effects of short-term salinity on lentil seedlings cultured in petri plates. Therefore, further investigation is required to evaluate whether l-Glu can mitigate the damages in lentil seedlings grown on salt-affected land.

## Figures and Tables

**Table 1:** Effect of l-Glutamic acid (l-Glu) on shoot fresh weight (SFW), shoot dry weight (SDW), water content (WC) and proline content (Pro) of lentil seedlings. The mean (± SE) was calculated from three replications. Values of different letters indicate statistically significant differences at *p* ≤ 0.05 (Fisher’s LSD test).

Treatments	SFW (mg shoot^−1^)	SDW (mg shoot^−1^)	WC (%)	Pro (µmol g^−1^ DW)
C	70.5 ± 2.3 a	12.1 ± 0.26 a	82.8 ± 0.5 b	24.5 ± 3.7 c
l-Glu	71.1 ± 1.8 a	11.8 ± 0.15 a	83.4 ± 0.5 a	28.4 ± 2.3 c
S	35.3 ± 1.4 c	9.4 ± 0.03 c	73.4 ± 1.0 d	116.3 ± 4.4 a
S + l-Glu	48.6 ± 1.9 b	9.97 ± 0.3 b	79.5 ± 0.3 c	67.7 ± 0.3 b

**Table 2:** Effect of l-Glutamic acid (l-Glu) on the level of leaf, shoot and root Na^+^, K^+^ and Na^+^/K^+^ ratio in lentil seedlings under salt stress and recovery conditions. The above mean (±SE) was calculated from three replications. Values of different letters indicate statistically significant differences at *p* ≤ 0.05 (Fisher’s LSD test).

	Treatment	Leaf	Shoot	Root
Na^+^ content (µmol g^−1^ DW)	C	9 ± 0.8 c	21 ± 0.6 c	20 ± 11 d
	l-Glu	21 ± 0.6 c	31 ± 1 c	145 ± 7 c
	S	1000 ± 28 a	1018 ± 22 a	1096 ± 14 a
	S + l-Glu	671 ± 26 b	691 ± 35 b	736 ± 45 b
K^+^ content (µmol g^−1^ DW)	C	315 ± 13 b	505 ± 11 a	377 ± 17 b
	l-Glu	342 ± 25 b	479 ± 9 a	489 ± 13 a
	S	524 ± 6 a	211 ± 14 c	206 ± 11 c
	S + l-Glu	539 ± 7 a	361 ± 8 b	344 ± 12 b
Na^+^/K^+^ ratio	C	0.03 ± 0.004 c	0.04 ± 0.002 c	0.06 ± 0.03 c
	l-Glu	0.06 ± 0.004 c	0.07 ± 0.001 c	0.3 ± 0.02 c
	S	1.9 ± 0.04 a	4.9 ± 0.4 a	5.4 ± 0.3 a
	S + l-Glu	1.3 ± 0.05 b	1.9 ± 0.1 b	2.2 ± 0.07 b

## Supplementary Materials

The following are available online at https://www.mdpi.com/article/10.3390/biom11040587/s1, Figure S1: Effect of different doses of l-Glutamic acid on lentil seedlings in response to salt stress and recovery condition, Figure S2: Effect of different doses of NaCl on lentil seedlings pretreatment with 10 mM l-Glu after 7 days of recovery condition, Figure S3: Effect of another amino acid in lentil seedlings under 110 mM NaCl stress and recovery condition, Figure S4: Effect of l-Glutamic acid on the level of (a) leaf Ca^2+^, (b) shoot Ca^2+^, (c) root Ca^2+^, (d) leaf Mg^2+^, (e) shoot Mg^2+^ and (f) root Mg^2+^ in lentil seedlings under salt stress and recovery condition, Figure S5: Correlation analysis between l-Glu and NaCl application and different growth attributes of lentil seedlings.
