# Supplementary material for: Modulation of the Antioxidant Defense System by Exogenous l-Glutamic Acid Application Enhances Salt Tolerance in Lentil (Lens culinaris Medik.)
Source: Biomolecules. 2021 Apr 16;11(4):587. doi: 10.3390/biom11040587 (PMC8073835; doi:10.3390/biom11040587)
Supplement: Supplementary file 1 [file biomolecules-11-00587-s001.zip › biomolecules-1172916-supplementary.pdf]

Supplementary Materials

Modulation of the Antioxidant Defense System by Exogenous L-Glutamic Acid Application Enhances Salt Tolerance in Lentil (*Lens culinaris* Medik.)

Jannatul Fardus, Md. Shahadat Hossain and Masayuki Fujita *

Laboratory of Plant Stress Responses, Faculty of Agriculture, Kagawa University, Ikenobe 2393, Miki-cho, Kita gun, Kagawa 761 0795, Japan

* Correspondence: fujita.masayuki@kagawa-u.ac.jp

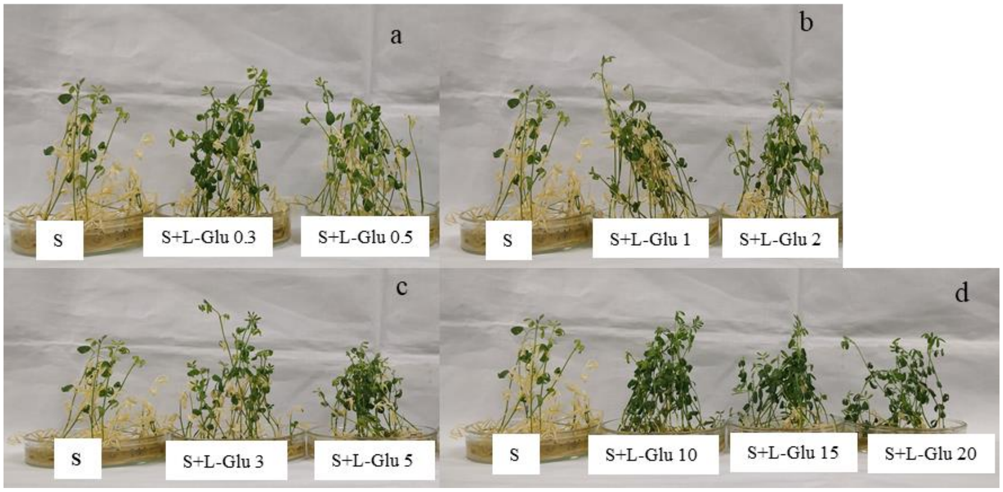

Figure S1. Effect of different doses of L-Glutamic acid on lentil seedlings in response to salt stress and recovery condition. (**a**) phenotypic appearance of lentil seedlings with pretreatment of 0.3 mM and 0.5 mM L-Glu after 7 days of recovery condition, (**b**) phenotypic appearance of lentil seedlings with pretreatment of 1 mM and 2 mM L-Glu after 7 days of recovery condition, (**c**) phenotypic appearance of lentil seedlings with pretreatment of 3 mM and 5 mM L-Glu after 7 days of recovery condition and (**d**) phenotypic appearance of lentil seedlings with pretreatment of 10 mM, 15 mM and 20 mM L-Glu after 7 days of recovery condition.

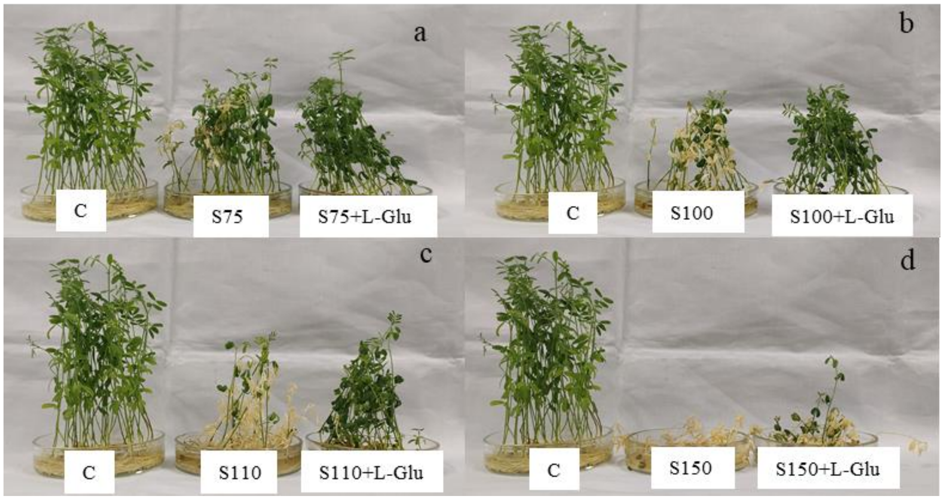

Figure S2. Effect of different doses of NaCl on lentil seedlings pretreatment with 10 mM L-Glu after 7 days of recovery condition. (**a**) phenotypic appearance of lentil seedlings with the application of 75 mM NaCl, (**b**) phenotypic appearance of lentil seedlings with the application of 100 mM NaCl, (**c**) phenotypic appearance of lentil seedlings with the application of 110 mM NaCl and (**d**) phenotypic appearance of lentil seedlings with the application of 150 mM NaCl.

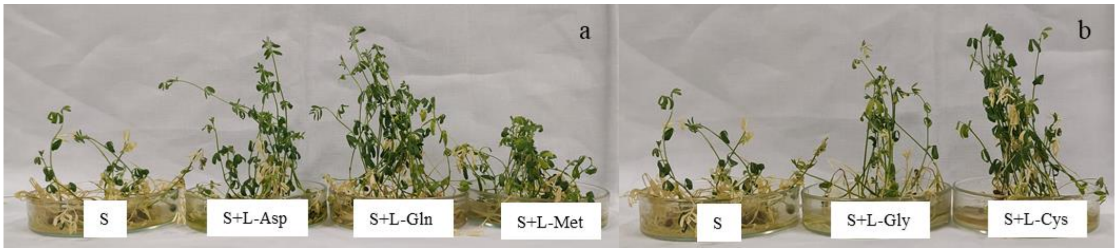

Figure S3. Effect of another amino acid in lentil seedlings under 110 mM NaCl stress and recovery condition. (**a**) phenotypic appearance of lentil seedlings with the pretreatment of 10 mM L-Asparagine (L-Asp), L-Glutamine (L-Gln) and L-Methionine (L-Met) after 7 days of recovery from salt stress condition, (**b**) phenotypic appearance of lentil seedlings with the pretreatment of 10 mM L-Glycine (L-Gly), and L-Cysteine (L-Cys) after 7 days of recovery from salt stress condition.

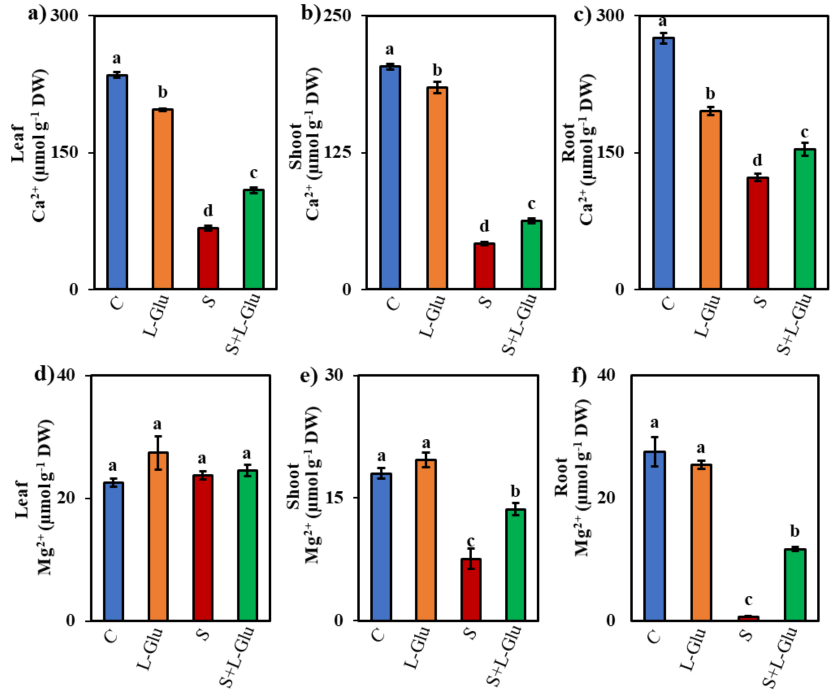

Figure S4. Effect of L-Glutamic acid on the level of (**a**) leaf Ca^{2+}, (**b**) shoot Ca^{2+}, (**c**) root Ca^{2+}, (**d**) leaf Mg^{2+}, (**e**) shoot Mg^{2+} and (**f**) root Mg^{2+} in lentil seedlings under salt stress and recovery condition. The above mean (± SE) was calculated from three replications. Values of different letters indicates statistically significant differences at $p \leq 0.05$ (Fisher's LSD test).

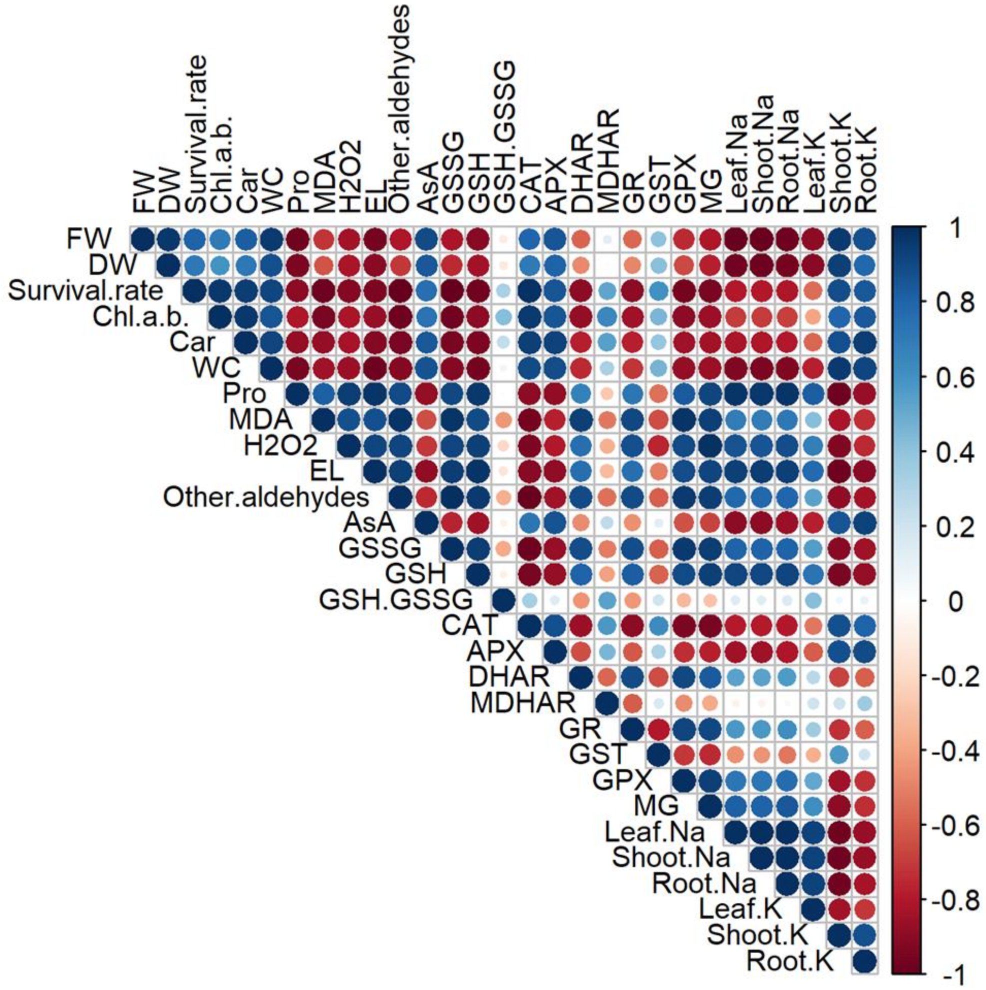

Figure S5. Correlation analysis between L-Glu and NaCl application and different growth attributes of lentil seedlings. FW (fresh weight of shoot), DW (dry weight of shoot), Survival. Rate (percentage of survivability), Chl.a.b (chlorophyll (a + b) content), Car (carotenoid content), WC (water content), Pro (proline content), MDA (malonaldehyde content), H_2O_2 (hydrogen peroxide content), EL (Electrolyte leakage percentage), Other. Aldehydes (other aldehyde content), AsA (ascorbate content), GSSG (oxidized glutathione content), GSH (reduced glutathione content), GSH.GSSG (Reduced glutathione and oxidized glutathione ratio), CAT (catalase activity), APX (ascorbate peroxidase activity), DHAR (dehydroascorbate reductase activity), MDHAR (monodehydroascorbate reductase activity), GR (glutathione reductase activity), GST (glutathione *S*-transferase activity), GPX (glutathione peroxidase activity), MG (methylglyoxal content), Leaf.Na (leaf Na^+ content), Shoot.Na (shoot Na^+ content), Root.Na (root Na^+ content), Leaf.K (leaf K^+ content), Shoot.K (shoot K^+ content), Root.K (root K^+ content).
